# Evaluating the effectiveness of integrating a clinical pharmacist into a hidradenitis suppurativa clinic within the specialty of dermatology

**DOI:** 10.1093/skinhd/vzag049

**Published:** 2026-04-29

**Authors:** Noemie Senawong, Kathleen Kroger, Damien Fisher

**Affiliations:** Department of Dermatology, University of Texas Medical Branch, League City, TX, USA; Department of Dermatology, University of Texas Medical Branch, League City, TX, USA; Department of Dermatology, University of Texas Medical Branch, League City, TX, USA

## Abstract

**Background:**

Ambulatory care pharmacists improve outcomes through medication management, adherence support, education and access to care. In dermatology, pharmacist integration is relatively new but may benefit patients with chronic conditions such as hidradenitis suppurativa (HS), which causes painful, inflammatory nodules and impaired quality of life.

**Objectives:**

To evaluate the impact of a dermatology pharmacist on outcomes in patients with HS.

**Methods:**

A prospective, quasiexperimental study was conducted in monthly HS clinics with a dermatology pharmacy specialist. The primary outcome was the Hidradenitis Suppurativa Quality of Life (HiSQOL) score, assessing symptoms, emotional wellbeing and functioning. Surveys were completed at baseline and at 3 and 6 months. Secondary outcomes included pharmacist interventions, categorized as medication facilitation, optimization and wound care.

**Results:**

In total, 38 patients were seen and evaluated by the clinical pharmacist. All completed baseline surveys; 30 (79%) completed the 3-month and 19 (50%) completed the 6-month follow-up. Mean HiSQOL scores improved from 19.8 at baseline to 12.7 at 3 months and 10.5 at 6 months. Paired *t*-tests showed significant reductions from baseline to 3 months (*P* < 0.001), from baseline to 6 months (*P* = 0.006) and from 3 months to 6 months (*P* = 0.03). Wilcoxon signed-rank tests confirmed significance across all intervals. In total 174 pharmacist interventions were recorded: medication facilitation (48.9%), medication optimization (36.8%) and wound care (14.4%).

**Conclusions:**

Incorporating a dermatology pharmacist in HS care significantly improves patients’ quality of life and provides critical support in medication access and optimization and wound care.

## Teaser

A prospective study at University of Texas Medical Branch found that incorporating a dermatology pharmacist into hidradenitis suppurativa clinic significantly improved patients’ quality of life, with Hidradenitis Suppurativa Quality of Life scores decreasing from 19.77 at baseline to 10.47 at 6 months. The pharmacist made 174 interventions, primarily focused on medication access, optimization and wound-care support.

What is already known about this topic?

- Hidradenitis suppurativa (HS) is a chronic inflammatory skin disease that causes painful inflammatory nodules, abscesses and draining tunnels.
- Complications include scarring, deformities, malodorous drainage and significant psychosocial burden.
- Patients often experience low self-esteem, depression and reduced work productivity.
- No cure exists; treatment focuses on symptom control and preventing progression.
- Management is complex and requires multidisciplinary care.

What does this study add?

- The results fill gaps in the limited data on pharmacists embedded in HS specialty clinics.
- A novel dermatology clinical pharmacy service was evaluated at University of Texas Medical Branch Health.
- We assessed the effects of pharmacist-led interventions on patient outcomes, such as symptoms, emotional wellbeing and functioning.
- Integration of a clinical pharmacist into an HS clinic is linked to significant improvements in patient-reported quality of life.

Hidradenitis suppurativa (HS) is a chronic inflammatory dermatological condition characterized by painful nodules and abscess formation in regions such as the axillae and groin.^1^ Painful lesions typically develop in intertriginous regions and areas with a high density of sweat glands.^2^ Due to its persistent and complicated inflammatory markers, HS frequently leads to complications, including scarring, limb contractures and malodorous drainage, all of which can substantially impair a patient’s quality of life, and contribute to both physical and psychosocial burdens.^3^ Affected individuals may experience diminished self-esteem, sexual health concerns, increased risks of depression and social isolation, and decreased work productivity.^4,5^

Currently, there is no available cure for this condition; therefore, the primary treatment objectives focus on preventing disease progression and alleviating symptoms.^6^ The management of HS is inherently complex and often necessitates a collaborative, multidisciplinary approach. Clinical ambulatory care pharmacists play a valued role in enhancing patient outcomes through facilitating medication access, monitoring adherence, optimizing therapeutic regimens and providing comprehensive patient education. In a study by Penick *et al.*, the addition of a pharmacist to the dermatology clinic significantly improved medication adherence from 93.1% to 99.2% and reduced the average time to initiation of medications from 21.3 to 13.6 days.^7^

Despite these promising findings, there are limited published data specifically evaluating the role of pharmacists embedded within an HS specialty clinic. To address this gap, a clinical pharmacy service was established at the University of Texas Medical Branch (UTMB) to assess the impact of a dermatology clinical pharmacist on the care of patients with HS. This study aimed to evaluate how pharmacist-led interventions influenced patient outcomes, particularly with respect to quality of life.

## Patients and methods

A prospective, quasiexperimental, cohort study was conducted from August 2024 to March 2025. Data were collected from the electronic medical records of patients who were going to be seen by the dermatology clinical pharmacy specialist within the specialty of dermatology at UTMB Health. The HS clinic was conducted every second Friday of the month at Victory Lakes Town Center.

Inclusion criteria consisted of adults aged ≥18 years with a documented diagnosis of HS, who had a referral to the dermatology clinic at Victory Lakes Town Center. Patients were excluded if they were not seen at the UTMB outpatient dermatology clinic or if they were diagnosed with another autoimmune disorder (e.g. rheumatoid arthritis, Crohn disease or ulcerative colitis).

During the visit, the clinical pharmacist played a vital role in patient education and care coordination. Guidance on vaccine administration was also provided to ensure patients were up to date with recommended immunizations, particularly prior to initiating immunosuppressive or immunomodulating therapies. The pharmacist counselled on injection training for self-administered biologics, and emphasized proper technique and safety. Furthermore, support for wound-care management was provided, including recommendations for appropriate wound-care supplies and education on hygiene practices to minimize infection risk. The pharmacist provided guidance on utilizing medication assistance programmes designed to enhance access to cost-intensive treatments and mitigate financial obstacles. The pharmacist coordinated with infusion services to ensure timely administration of infused therapies, while collaborating with dermatologists and other specialists to support comprehensive, multidisciplinary care tailored to the specific needs of patients with HS.

The primary outcome was the score from the Hidradenitis Suppurativa Quality of Life (HiSQOL) survey, a validated questionnaire for the assessment of HS-specific quality of life. The questionnaire is used in clinical research and practice to subjectively evaluate HS-related symptoms, emotional and psychosocial impact, and how the condition affects daily activities. Each item is formatted as a Likert-type scale with either five or six response options, and items are scored from 0 (not at all) to either 4 (extremely) or 5 (unable to do). An average score was calculated for each category by summing the responses and dividing by the number of patients who completed the HiSQOL survey. Patients completed HiSQOL surveys during their initial visit with the clinical pharmacist, with additional surveys conducted at 3- and 6-month follow-up appointments to assess changes over time. Additional outcomes gathered included types of pharmacist interventions divided into three categories: medication facilitation, medication optimization and wound care.

To evaluate changes in HiSQOL scores over time, parametric and nonparametric paired statistical tests were employed. Paired *t*-tests were used to compare mean HiSQOL scores between baseline and 3 months, baseline and 6 months, and 3-month and 6-month timepoints for patients with available paired data. Given the small sample sizes and potential for non-normal distribution of patient-reported outcome scores, Wilcoxon signed-rank tests were also conducted as a nonparametric alternative to validate the findings. Statistical significance was defined as a *P*-value <0.05. These analyses were used to assess whether changes in HiSQOL scores over time were statistically meaningful, reflecting improvements in quality of life among patients engaged in pharmacist-led care.

## Results

During the study period, 38 patients were seen and evaluated by the clinical pharmacist. The mean (SD) age of the cohort was 35.1 (17.6) years, and the majority of the study population were women (*n* = 25/38; 66%) (Table 1). The most common reported ethnicity was Hispanic (*n* = 14/38; 37%), followed by Black (*n* = 13/38; 34%). Median body mass index was 36.5 kg m^−2^ (interquartile range 15.2–58.7). Half of the patients (*n* = 19; 50%) had medical insurance.

**Table 1:** Baseline cohort characteristics of 38 patients evaluated by the dermatology clinical pharmacy specialist in the hidradenitis suppurativa clinic

Characteristic	*n* (%)
Female sex	25 (66)
Age (years), mean (SD)	35.1 (17.6)
Race and ethnicity	
Black	13 (34)
Hispanic	14 (37)
White	10 (26)
Asian	1 (3)
Body mass index (kg m^−2^), median ­(IQR)	36.5 (15.2–58.7)
Insurance status	
Medical insurance	19 (50)
Medicare	3 (8)
Medicaid	13 (34)
No health coverage	3 (8)
History of type 2 diabetes mellitus	10 (26)
Hurley stage	
I	2 (5)
II	6 (16)
II–III	2 (5)
III	28 (74)
HiSQOL survey completion	
Baseline	38 (100)
3 months	30 (79)
6 months	19 (50)

Data are presented as *n* (%) unless otherwise specified. HiSQOL, Hidradenitis Suppurativa Quality of Life; IQR, interquartile range.

A history of type 2 diabetes mellitus was present in 10 of 38 patients (26%). Hurley staging of disease severity showed that the majority of patients (*n* = 28/38; 74%) had stage III HS.

Of the 38 patients evaluated by the clinical pharmacist, all (100%) completed an initial HiSQOL survey, 30 (79%) completed a 3-month survey and 19 (50%) completed a 6-month survey (Table 2).

**Table 2:** Patient-reported quality-of-life improvements based on Hidradenitis Suppurativa Quality of Life (HiSQOL) scores at baseline, 3 months and 6 months

Comparison	*n* (%)	Mean score (earlier → later)	Paired *t*-test *P*-value	Wilcoxon signed-rank *P*-value
Baseline vs. 3 months	30 (79)	19.8 → 12.7	<0.001	<0.001
Baseline vs. 6 months	19 (50)	18.2 → 10.5	0.006	0.003
3 months vs. 6 months	18 (47)	12.7 → 9.9	0.03	0.01

Percentages are calculated based on the total baseline cohort (*n* = 38). Analyses include only patients with paired HiSQOL data available at both timepoints for each comparison.

The mean HiSQOL score decreased from 19.8 at baseline to 12.7 at 3 months, and further decreased to 10.5 at 6 months. Similarly, for the subset of patients with both 3- and 6-month scores (*n* = 18/38; 47%), mean scores improved from 12.7 to 9.9.

Paired *t*-tests showed statistically significant reductions in HiSQOL scores from baseline to 3 months (*P* < 0.001), from baseline to 6 months (*P* = 0.006) and from 3 months to 6 months (*P* = 0.03). These findings were corroborated by nonparametric Wilcoxon signed-rank tests, which also demonstrated statistically significant improvements across all timepoints: baseline to 3 months (*P* < 0.001), baseline to 6 months (*P* = 0.003) and 3 months to 6 months (*P* = 0.01).

In total, 174 pharmacist interventions were made among the 38 patients seen, for an average intervention rate of approximately 4 interventions per patient (Table 3). Types of pharmacist intervention included medication facilitation (*n* = 85/174; 48.9%), medication optimization (*n* = 64/174; 36.8%) and wound care (*n* = 25/174; 14.4%).

**Table 3:** Description of 174 clinical pharmacist interventions among 38 patients at the hidradenitis suppurativa clinic

Intervention	*n* (%)^a^
Medication facilitation	85 (48.9)
Medication initiation	
Medication assistance (PAP or HSW)	
Refill management	
Medication optimization	64 (36.8)
Dose adjustments	
Lab monitoring	
Patient education and injection device training	
Medication adherence	
Wound care	25 (14.4)
Hygiene practices	
Wound-care supplies	

HSW, hardship waiver; PAP, prescription assistance programme. ^a^The data reflect the total number of interventions documented among 38 patients. Missing data from 3- and 6-month follow-ups are acknowledged but not individually represented in this table.

## Discussion

Our study assessed the clinical impact after the addition of a dermatology clinical pharmacy specialist to the UTMB HS clinic. Descriptive and inferential statistics were calculated using the appropriate tests based on data type and number of groups. This study demonstrates that the integration of a clinical pharmacist into an HS clinic is associated with significant improvements in patient-reported quality of life, as measured by HiSQOL scores. The average HiSQOL score decreased from baseline to 3 months then further decreased at 6 months, indicating meaningful improvement in disease burden over time. These findings were statistically significant in paired *t*-tests and nonparametric Wilcoxon signed-rank tests across all intervals, reinforcing the reliability of the observed trend.

The study population was predominantly female and Hispanic, and a high proportion had severe disease. Many patients also presented with significant comorbidities, including obesity and type 2 diabetes mellitus, which underscores the complexity of care required in this population. The improvement in HiSQOL scores despite these factors suggests that comprehensive, pharmacist-led interventions may play a meaningful role in addressing unmet needs in HS care.

Throughout the study, nearly half of the interventions made by the clinical pharmacist involved medication access support, which is a known barrier to the care in the specialty of dermatology, particularly among underserved populations. Comparable findings were reported in a previous study, which showed that incorporating a pharmacist into a dermatology clinic setting reduced the time to medication initiation.^7^ Medication optimization and wound-care guidance comprised the remainder of the interventions, which reflects the multifaceted role of the pharmacist in treatment education, disease management and coordination of care.

HiSQOL was newly implemented in this dermatologist–clinical pharmacist collaborative clinic, with assessments administered by the clinical pharmacist, as the physician did not routinely collect disease-specific quality of life measures. No comparator data were available due to the clinic’s recent establishment. While unmeasured confounders may have influenced score changes, HiSQOL supported the clinical value of clinical pharmacist integration, and future studies may enable interclinician comparisons and adjustment for confounders.

Our study had several limitations. A larger study sample would have aided in detecting small differences between the groups. Additionally, adherence to prescribed treatments could not be consistently verified, particularly for patients who filled their medications at pharmacies outside of the UTMB health system. This lack of adherence monitoring introduces uncertainty regarding the true extent of the pharmacological interventions received. Furthermore, HiSQOL scores were not available for patients lost to follow-up, which may introduce attrition bias and potentially overestimate improvements if patients with less favourable outcomes were less likely to complete follow-up assessments.

In conclusion, HS is a chronic, debilitating skin condition that severely affects patients’ quality of life and psychosocial health. This study highlights the meaningful role of a dermatology clinical pharmacy specialist in a multidisciplinary HS clinic and demonstrates their positive impact on patient outcomes. Improvements in HiSQOL survey scores over time suggest that pharmacist involvement contributes to better disease management and enhanced quality of life. In addition to supporting medication access and optimization, pharmacists play a critical role in wound-care education, which may help prevent infections and reduce complications associated with HS. These educational efforts are crucial in empowering patients to take an active role in their treatment and self-care routines, ultimately leading to better disease control and fewer hospitalizations or emergency visits. Moreover, their involvement can foster stronger patient–provider relationships, increasing adherence to treatment plans and addressing concerns promptly.

These findings underscore the importance of including clinical pharmacists as integral members of the healthcare team to effectively manage the complex needs of patients with HS. Incorporating pharmacists into routine care not only improves clinical outcomes but also supports the emotional and psychological resilience of patients by addressing the often-overlooked aspects of living with a chronic skin condition.
